# Protracted Neural Development of Dorsal Motor Systems During Handwriting and the Relation to Early Literacy Skills

**DOI:** 10.3389/fpsyg.2021.750559

**Published:** 2021-11-19

**Authors:** Sophia Vinci-Booher, Karin H. James

**Affiliations:** Department of Psychological and Brain Sciences, Indiana University, Bloomington, IN, United States

**Keywords:** handwriting, fMRI, dorsal visual stream, literacy, development

## Abstract

Handwriting is a complex visual-motor skill that affects early reading development. A large body of work has demonstrated that handwriting is supported by a widespread neural system comprising ventral-temporal, parietal, and frontal motor regions in adults. Recent work has demonstrated that this neural system is largely established by 8 years of age, suggesting that the development of this system occurs in young children who are still learning to read and write. We made use of a novel MRI-compatible writing tablet that allowed us to measure brain activation in 5–8-year-old children during handwriting. We compared activation during handwriting in children and adults to provide information concerning the developmental trajectory of the neural system that supports handwriting. We found that parietal and frontal motor involvement during handwriting in children is different from adults, suggesting that the neural system that supports handwriting changes over the course of development. Furthermore, we found that parietal and frontal motor activation correlated with a literacy composite score in our child sample, suggesting that the individual differences in the dorsal response during handwriting are related to individual differences in emerging literacy skills. Our results suggest that components of the widespread neural system supporting handwriting develop at different rates and provide insight into the mechanisms underlying the contributions of handwriting to early literacy development.

## Introduction

Handwriting is an important sensorimotor skill that takes years to develop. Most children begin their experience with handwriting by producing individual letters of the alphabet by kindergarten, yet the fluid and efficient production of letter-forms in the creation of words and complex sentences continues to develop throughout middle school (Feder et al., 2000; Treiman and Kessler, 2014; Coker and Ritchey, 2015; Mangen and Balsvik, 2016; Fears and Lockman, 2018). Thus, the earliest and most fundamental element of handwriting is the coordination of sensory and motor systems to produce a written form, a task not discernible from drawing. Indeed, neural responses found during handwriting in literate adults are extremely similar to those found during drawing, with only minor differences found in the parietal cortex (Yuan and Brown, 2014, 2015; Ose Askvik et al., 2020). Understanding the development of the sensorimotor system supporting handwriting – how it differs between adults and young children at the earliest stages of learning to write – can provide valuable insights into the role of sensorimotor systems in learning.

Much of what we know about how this neural system supports handwriting comes from studies on adult populations. The adult literature on handwriting suggests that handwriting is supported by a largely left-lateralized neural system comprised of ventral-temporal, parietal, and frontal motor regions (Katanoda et al., 2001; Beeson et al., 2003; James and Gauthier, 2006; Purcell et al., 2011; Rapp and Dufor, 2011; Dufor and Rapp, 2013; Planton et al., 2013, 2017; Yuan and Brown, 2014, 2015; Longcamp et al., 2014; Vinci-Booher et al., 2019; Vinci-Booher and James, 2020b). The involvement of brain regions in this broad neural system has been related to different aspects of the handwriting experience. For example, studies have been conducted to determine which brain regions were related to the sensorimotor action of handwriting and which regions were related to other processes that are commonly engaged during handwriting tasks, such as spelling (Planton et al., 2017). A recent meta-analysis of such studies found that the sensorimotor element of handwriting was primarily supported by the left parietal and frontal cortices (Purcell et al., 2011). The authors noted that additional cortical regions may also be related to the sensorimotor element that were not identified in their meta-analysis, because many of the studies included in their meta-analysis explicitly controlled for the sensorimotor element.

Our prior work in adults evaluated the degree to which the brain regions associated with the sensorimotor element of handwriting could be separated into sensorimotor components, namely, motor and visual components (Vinci-Booher et al., 2019). The motor component was isolated by comparing activation during handwriting to activation while participants passively watched a letter unfold as if being written. The visual component was isolated by comparing activation during handwriting to activation during handwriting using a pen that had no ink. We found that a largely left-lateralized neural system comprised of ventral-temporal, parietal, and frontal motor regions was recruited during handwriting and that the response in frontal motor and parietal regions was related to the motor component of handwriting (i.e., producing the letter), similar to the results of the meta-analysis (Purcell et al., 2011). Our results added, however, that a ventral response was also apparent during handwriting and that this ventral response was related to the visual component of handwriting (i.e., perceiving the letter as it is produced). Of note was an area of motor-visual overlap where activation appeared to be equally related to the motor and visual component of handwriting: the left intraparietal sulcus (Vinci-Booher et al., 2019).

Prior work on the development of the neural system supporting handwriting is limited but generally indicates that the adult neural system is largely in place by the middle school years (i.e., by approximately 11–13 years of age) and perhaps a few years earlier. Work with typically developing middle school children using EEG found that handwriting, drawing, and typing produced reliable differences in brain oscillations in adults that were also observed in middle school children, suggesting that neural processing during handwriting was already adult-like in middle school children (Ose Askvik et al., 2020). Work in clinical populations using fMRI has demonstrated that neural responses during handwriting in middle school children that deviated from the adult-like response were associated with dysgraphia and/or dyslexia, suggesting that the onset of an adult-like neural response during handwriting by the middle school years is associated with typical development (Richards et al., 2011, 2015, 2017). Additionally, one recent study suggested that the neural system that supports handwriting might even be in place prior to middle school (Palmis et al., 2021). In this study, children ages 8–11 years of age and adults were asked to write on a touchscreen tablet during fMRI scanning (Tam et al., 2011; Longcamp et al., 2014). Results demonstrated no significant differences between children and adults in whole brain activation patterns, suggesting that the spatial topography of regions involved in handwriting may be adult-like by as early as 8 years of age.

The development of the neural system supporting handwriting in elementary school children younger than 8 years old is currently unknown; however, hypotheses concerning its development can be made from considering general developmental trends. At least three lines of research suggest that processing in the dorsal cortex, namely, parietal cortex, undergoes a protracted developmental trajectory relative to the ventral-temporal cortex when investigated past 2 years of age (Dekker et al., 2011; Stiles et al., 2013; Freud et al., 2016, 2019; Vinci-Booher and James, 2020a; Vinci-Booher et al., in press). First, behaviors that are often associated with neural processing in the posterior parietal cortex were not yet adult-like by 4.5–6.5 years of age while behaviors associated with processing in the ventral-temporal cortex were adult-like (Freud et al., 2019). Second, the tissue properties of major white matter tracts that connect parietal and frontal motor cortices were not yet adult-like in 5–8-year-old children while white matter tracts predominantly contained within the ventral-temporal cortex were adult-like (Lebel et al., 2008; Stiles et al., 2013; Vinci-Booher et al., in press). Finally, studies using children of approximately the same age ranges have found that object selectivity develops later in the parietal cortex than in ventral-temporal cortex for tools and animals (Dekker et al., 2011) and letters (Vinci-Booher and James, 2020a). Together, these three lines of work suggest that young children rely on different neural systems than adults. More specifically, they suggest that parietal involvement during handwriting may still be developing in elementary school children younger than 8 years of age.

We hypothesized that the responses of brain regions within the neural system supporting handwriting in children younger than 8 years of age would be different from its response in adults. Given the substantial evidence in support of a protracted development of the parietal cortex in young children (e.g., Stiles et al., 2013; Freud et al., 2016; Vinci-Booher et al., in press), we expected that parietal function during handwriting would still be developing in typically developing children younger than 8 years of age. We also expected that the response in the handwriting neural system would be related to early reading development. Studies of handwriting development in children younger than 8 years old have demonstrated that handwriting experience increases activation in several regions that come to support the perception of letters of the alphabet (James, 2010; James and Engelhardt, 2012; Kersey and James, 2013; Vinci-Booher et al., 2016), suggesting that handwriting is influential in neural changes associated with learning to read.

Investigating the development of brain regions supporting handwriting in children younger than 8 years of age comes with several challenges. First, young children are prone to movement and movement presents difficulty for MRI data. Our lab specializes in collecting MRI data from young children even while performing an in-scanner task (e.g., James and Kersey, 2018), including procedures for reducing motion during the scan and for properly addressing motion when it does occur. Second, young children experience extreme difficulty writing letters using the MRI-compatible writing tablets that are currently available because they are unable to see their hand when they are writing (Mraz et al., 2004; Rektor et al., 2006; Tam et al., 2011; Reitz et al., 2013; Karimpoor et al., 2015; Ko et al., 2018). The inability to see their hand during writing makes it very difficult for young children to write letters because they have not yet developed the competence seen in adults and older children who have substantially more practice writing letters of the alphabet (unpublished data). We, therefore, developed an MR-compatible writing tablet, the MRItab (Vinci-Booher et al., 2018). The MRItab is a touchscreen tablet with a video display that provides the user with an experience similar to the common smartphone or tablet. Importantly, the MRItab makes it feasible for young children to write during fMRI scanning because participants can see their hand when they are writing.

To better understand the developmental trajectory of the neural system supporting handwriting and its relationship to early reading development, we assessed neural activation using fMRI imaging in adults and 5–8-year-old children while they wrote letters to dictation. We focused on activation in regions of the ventral-temporal, parietal, and frontal motor cortices that have been identified as being involved with handwriting in adults (Katanoda et al., 2001; Beeson et al., 2003; James and Gauthier, 2006; Purcell et al., 2011; Planton et al., 2013, 2017; Longcamp et al., 2014; Yuan and Brown, 2014, 2015; Vinci-Booher et al., 2019). All participants wrote letters to dictation on the MRItab with a writing utensil. In one condition, they saw what they wrote as they were writing (Write Ink), in a second condition, they wrote with a pen that had “no ink” (Write No Ink), and in a third condition, they observed a letter unfolding as if being written to dictation (Watch Ink). The latter two conditions were designed to allow us to observe activations during two components of handwriting: the motor component during the Write No Ink condition, that is the hand movements required to create the letter, and the visual component during the Watch Ink condition, that is seeing the letter-form unfold as if being written. We also evaluated the relationships between the neural responses in each ROI and a literacy composite score to determine the relationship between the development of the neural system supporting handwriting and emerging literacy.

## Materials and Methods

### Participants

All participants were recruited through word of mouth or an in-house database of community members. Adult participants provided written informed consent and were compensated monetarily for their time. Parents of all children provided written informed consent and children who were 7 years and older provided written informed assent. Participating families were compensated for their time with gift cards as well as small toys for the children. All participants were right-handed, expressed English as their native language, and were free of neurological trauma, developmental disorders, and MRI contraindications.

We obtained usable data from 14 adults and 27 children after excluding five children due to difficulty following instructions and/or technical difficulties as well as nine children and three adults due to an unacceptable amount of motion during conditions of interest. The 13 youngest children were assigned to the younger age group (M = 5.5 years, SD = 0.5 years; seven females, six males) and the 14 oldest children were assigned to the older age group (M = 7.6 years, SD = 0.5 years; eight females, six males). The children were separated into younger and older age groups for consistency with prior work that incorporated data from these same participants (Dekker et al., 2011; Stiles et al., 2013; Freud et al., 2016, 2019; Vinci-Booher and James, 2020a; Vinci-Booher et al., in press). The adult sample included 14 adults (M = 20.31, SD = 1.02; nine females, five males). Sample sizes were selected in line with prior work (Dekker et al., 2011; Vinci-Booher and James, 2020a); *post hoc* power is reported at alpha equal to 0.05.

### Materials and Stimuli

#### Apparatus

**FIGURE 1:**
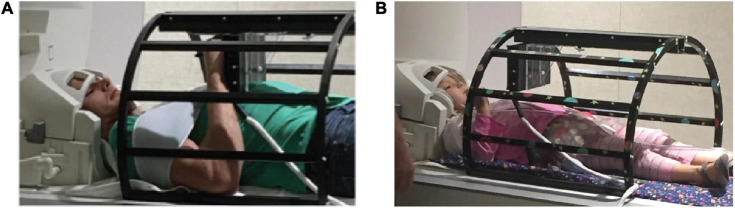
Experimental set up. Adult and child participants (**A,B**, respectively) completed all tasks using a novel MR-compatible writing tablet, the MRItab. The MRItab was mounted to a holding apparatus and positioned at a location that allowed the participant to see and interact with the tablet easily.

Participants used the MRItab for all conditions (Vinci-Booher et al., 2018; Figure 1). The MRItab is an MR-compatible digital tablet with touchscreen and display capabilities that provides a user experience similar to writing on a common smart-phone or tablet. The MRItab was affixed above each participate through a mounting system. To reduce motion, all participants wore a Wheaton^®^ elastic shoulder immobilizer and inflatable padding was used for padding between participants’ heads and the head coil. Verbal instructions were delivered through MRI-compatible headphones. An in-house Matlab program using the Psychophysics Toolbox extensions interfaced with the headphones, projector, and either tablet to record and present all stimuli (Brainard, 1997; Pelli, 1997).

#### Stimuli

Twelve letters from the Roman alphabet were selected: A, B, C, D, G, H, J, L, Q, R, U, and Y. Based on pilot testing, we expected our youngest children to know and be able to write the 12 letters to dictation within a 4 s time frame. This set of 12 letters also reduces the use of easily confusable letter names (e.g., c and e). All letters were written in white on a black background with a pen width of 7 points within a box that subtended 10 by 10 degrees of visual angle.

### Procedure

#### MRI Procedure

All children underwent training in a mock MRI scanner before entering the MRI environment; adults did not undergo training in a mock MRI scanner. The training was necessary to ensure the ability of each child participant to perform the handwriting task and to ensure that they understood and were able to stay still during the experiment. Only children who produced letter-like forms during the mock training session and appeared comfortable in the mock MRI environment continued in the study. A trained research assistant always remained in the MRI room with the child to provide support and help the child stay on-task. A second trained research assistant ran the experimental protocol from the MRI operator room while watching the child on a camera to ensure that they were on-task during all conditions. Except for the mock MRI training session and the presence of an additional research assistant, the procedures for the children and adults were the same.

All participants underwent a high-resolution anatomical scan followed by up to four fMRI experimental runs, depending upon the comfort and compliance of the participant. During the anatomical scan, participants watched a movie, listen to an audiobook, or simply rested. Each functional run contained one complete set of experimental conditions and lasted 344 s (5:44 min) [see Figure 2 in Vinci-Booher and James (2020a)]. Block orders were pseudo-randomized and counter-balanced across participants.

Each block of the functional runs contained six 4-s trials; one stimulus was presented in each of the six trials. Blocks were separated by 14-s inter-block intervals. During the inter-block interval, only the fixation cross was visible in the mirror. The last 2 s of each inter-block interval contained auditory instructions for the following block: “draw” or “watch.”

Each trial began with an auditory prompt that indicated the letter for that trial (e.g., “A” or “B”). During Write Ink and Write No Ink trials, the participant wrote this letter using an MR-compatible pen. In the Write Ink condition, they saw their letter being produced as they wrote it. In the Write No Ink condition, no trace was left from their pen as if their pen had “no ink.” During Watch Ink trials, participants passively watched a video of their own letter production unfold as if it were being written. The pen trajectory that they watched was a pen trajectory of their own letter production that had been recorded. In all conditions, the screen became blank at the end of each 4 s trial, and a new letter was prompted.

#### Behavioral Procedure

All participants that successfully completed the MRI scanning session were asked to return for a one-hour behavioral session with the requirement that the behavioral session must occur within 3 weeks of the neuroimaging session. The behavioral session consisted of a battery of standard assessments designed to assess visual-motor integration, fine motor skill, and literacy level. Only literacy assessments were of interest in the current study. Literacy assessments included four subtests from the Woodcock Johnson IV Tests of Achievement: Letter-Word Identification, Spelling, Word Attack, Spelling of Sounds (Schrank and Wendling, 2018). These four literacy assessments were averaged to create a composite literacy score. All participants completed the assessments; however, only the scores from the children were of interest in the current study. Group means and standard errors for each literacy assessment and the composite literacy score are reported in Table 1.

**TABLE 1:** Mean and standard deviation of behavioral assessments.

	Younger Children (*n* = 13)	Older Children (*n* = 14)	Children (*n* = 27)
	M (SD)	M (SD)	M (SD)
Age (years)	5.5 (0.6)	7.7 (0.5)	6.6 (1.2)
**Woodcock Johnson IV**			
Letter Word Identification	22.4 (14.2)	50.5 (16.3)	37.0 (20.8)
Spelling	9.7 (2.5)	23.3 (8.9)	16.7 (9.5)
Word Attack	9.7 (4.9)	21.4 (5.0)	15.7 (7.7)
Spelling of Sounds	6.4 (2.9)	15.2 (4.4)	11.0 (5.8)
Literacy Composite Score	12.0 (5.7)	27.6 (8.0)	20.1 (10.5)

### MRI Scanning Parameters

Neuroimaging was performed at the Indiana University Imaging Research Facility, housed within the Department of Psychological and Brain Sciences with a Siemens Prisma 3-T whole-body MRI system. High-resolution T1-weighted anatomical volumes were acquired using an MPRAGE sequence: TI = 900 ms, TE = 2.98 ms, TR = 2300 ms, flip angle = 9°, with 176 sagittal slices of 1.0 mm thickness, a field of view of 256 × 248 mm, and an isometric voxel size of 1.0 mm^3^. For functional images, the field of view was 220 × 220 mm, with an in-plane resolution of 110 × 110 pixels and 72 axial slices of 2.0 mm thickness per volume with 0% slice gap, producing an isometric voxel size of 2.0 mm^3^. Functional images were acquired using a gradient-echo EPI sequence with interleaved slice order: TE = 30 ms, TR = 1000 ms, flip angle = 52° for blood-oxygen-level-dependent (BOLD) imaging.

### MRI Data Processing

#### Preprocessing

All MRI data preprocessing was performed using BrainVoyager QX, Version 2.8 (Brain Innovation) and was performed as previously reported in Vinci-Booher and James (2020a). The preprocessing steps will be reiterated here: Preprocessing of functional data included slice scan time correction, 3-D motion correction using trilinear/sinc interpolation, and 3D Gaussian spatial blurring with a full-width at half-maximum of 6 mm. Temporal high-pass filtering was performed using a voxel-wise GLM with predictors that included a Fourier basis set with a cut-off value of 2 sine/cosine pairs and a linear trend predictor. To account for head motion, we calculated the relative root mean squared (RMS) time course for each run using the rigid transformation parameters and counted the number of timepoints within a functional run with RMS > 2.0 mm (Van Dijk et al., 2012; Satterthwaite et al., 2013). If this number was greater than or equal to seven, the entire run was removed from the analysis. Additionally, if visual inspection of the rigid body motion parameters indicated a large amount of non-spiking motion in at least one parameter, the entire run was removed from the analysis. This procedure resulted in a final dataset of 13 younger children, 14 older children, and 14 adults, from sample sizes of 17, 19, and 18, respectively. Individual anatomical volumes were normalized to Talairach space (Talairach and Tournoux, 1988). Coregistration of functional volumes to anatomical volumes was performed using a rigid body transformation. Region of interest (ROI) analyses were performed using the peak percent BOLD signal change from anatomically localized 10 mm^3^ ROIs during the Write Ink, Write No Ink, and Watch No Ink conditions.

#### Anatomical ROI Placement

Individual participant ROIs were placed based on their anatomical image in Talairach space. Anatomical locations were determined by, first, referencing the Talairach Daemon and, second, confirming the location by referencing the human brain atlas to verify. Two ROIs were placed in the ventral temporal cortex: the left anterior fusiform gyrus (LaFuG) and the left posterior fusiform gyrus (LpFuG). Three ROIs were placed along the intraparietal sulcus in parietal cortex: the left anterior intraparietal sulcus (LaIPS), the left middle intraparietal sulcus (LmIPS), and the left posterior intraparietal sulcus (LpIPS). Two ROIs were placed in the frontal motor cortex: the left dorsal precentral gyrus (LdPG) and the left ventral precentral gyrus (LvPG).

### Statistical Analyses

#### ROI Analyses

We were primarily interested in understanding if the neural responses in specific regions of the ventral-temporal, parietal, and frontal motor cortices during handwriting changed with age. While a whole brain analysis might increase the likelihood of finding brain regions to be active during handwriting that were outside of our cortical areas of interest [e.g., the cerebellum (Purcell et al., 2011; Planton et al., 2013)], we chose to restrict our analyses to anatomically specific ROIs that we selected based on *a priori* hypotheses concerning their involvement in handwriting. Additionally, ROI analyses are more powerful and more robust against motion-related artifacts than other statistical analyses (e.g., functional connectivity; Poldrack, 2007).

**FIGURE 2:**
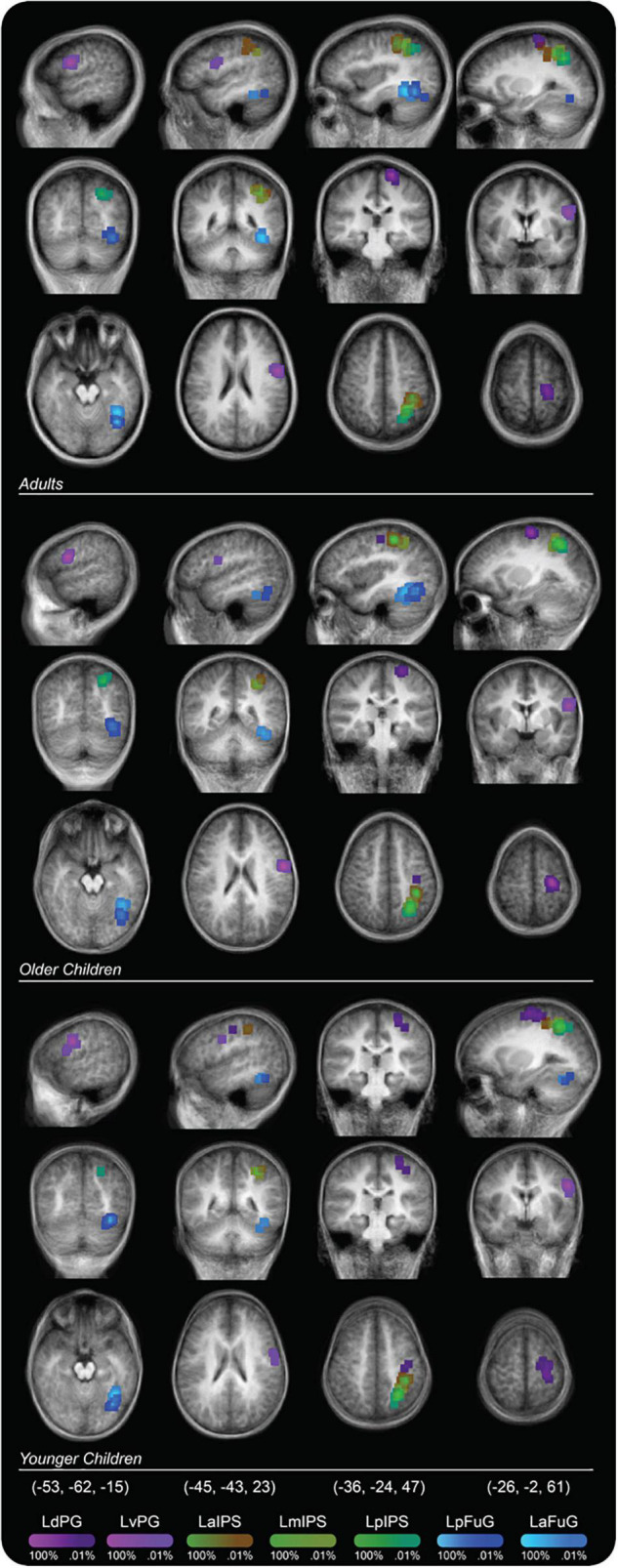
Probability map for regions of interest (ROIs) displayed on a group averaged anatomical image. Percent values correspond to the percentage of participants in a particular group whose ROI placement included that voxel.

The ROIs within each region were selected based on prior works that indicated potential involvement of these regions with handwriting (Katanoda et al., 2001; Beeson et al., 2003; James and Gauthier, 2006; Purcell et al., 2011; Planton et al., 2013, 2017; Longcamp et al., 2014; Yuan and Brown, 2014, 2015; Vinci-Booher et al., 2019). For the ventral-temporal cortex, ROIs included the left anterior fusiform gyrus (LaFuG) and the left posterior fusiform gyrus (LpFuG). For the parietal cortex, the ROIs included the left anterior intraparietal sulcus (LaIPS), left middle intraparietal sulcus (LmIPS), and left posterior intraparietal sulcus (LpIPS). For the frontal motor cortex, the ROIs included the left dorsal precentral gyrus (LdPG) and the left ventral precentral gyrus (LvPG). Probability maps for each ROI are shown in Figure 2 and the mean and standard deviation of the Talairach coordinates for each ROI are reported in Table 2.

**TABLE 2:** Mean and standard deviation of Talairach coordinates for ROIs.

Participant Group	ROI	Mean			Standard		
					Deviation		
		x	y	z	x	y	z
Adults	LdPG	−18.4	–24.3	64.8	2.6	5.2	3.2
	LvPG	−52.2	0.5	25.3	3.0	2.9	2.2
	LaIPS	−36.1	–35.4	46.6	4.3	2.7	5.1
	LmIPS	−31.6	–47.5	45.86	4.5	4.0	4.5
	LpIPS	−28.6	–57.1	41.1	3.9	3.4	3.2
	LpFuG	−36.1	–58.9	–13.2	2.7	4.4	3.4
	LaFuG	−37.3	–46.3	–13.2	1.9	3.9	3.4
Older Children	LdPG	−23.8	–18.4	60.3	4.0	3.2	3.2
	LvPG	−53.6	1.6	22.3	2.5	1.7	2.9
	LaIPS	−33.4	–34.6	49.9	2.2	2.9	2.6
	LmIPS	−27.4	–49.4	48.2	2.8	2.9	3.3
	LpIPS	−25.9	–56.0	45.8	2.5	3.1	2.8
	LpFuG	−35.6	–61.5	–15.5	2.6	4.1	4.3
	LaFuG	−36.9	–49.0	–16.1	2.4	4.4	4.7
Younger Children	LdPG	−28.6	–18.8	58.4	5.6	6.3	5.5
	LvPG	−52.7	–2.4	28.7	2.2	3.3	4.5
	LaIPS	−35.2	–35.6	49.1	3.3	2.8	2.7
	LmIPS	−29.4	–49.5	48.0	2.2	3.8	2.7
	LpIPS	−25.4	–55.8	44.6	2.3	4.2	2.7
	LpFuG	−36.2	–50.6	–17.2	3.4	3.2	3.2
	LaFuG	−34.5	–61.7	–17.0	3.5	3.8	3.0

*Units are in 1 mm isometric voxels. LdPG, left dorsal precentral gyrus; LvPG, left ventral precentral gyrus; LaIPS, left anterior intraparietal sulcus; LmIPS, left middle intraparietal sulcus; LpIPS, left posterior intraparietal sulcus; LpFuG, left posterior fusiform gyrus; LaFuG, left anterior fusiform gyrus.*

For each ROI, we performed a Two-way Repeated Measures ANOVA, with age group and writing condition as factors. The age group factor had three levels: younger children, older children, and adults. The writing condition factor had three levels: Write with Ink, Write No Ink, and Watch Ink. The dependent variable for all ANOVAs was peak percent BOLD signal change. Values that were greater or less than 3 standard deviations of the within-ROI, within-group mean were removed. As these comparisons were considered *a priori* comparisons, the results of the ANOVA analyses were considered significant based on uncorrected *p*-values. We note, however, that several comparisons would have passed Bonferonni correction for 7 comparisons, i.e., 7 ROIs, at a threshold of *p_bonferroni_* = 0.05/7 = 0.007. Simple effects analyses (One-way Repeated Measures ANOVAs) were performed following significant two-way interactions to compare activation among writing conditions within each age group and were followed with three *a priori* paired samples *t*-tests within each age group: Write Ink vs. Write No Ink, Write Ink vs. Watch Ink, and Write No Ink vs. Watch Ink. All *p*-values are reported as uncorrected *p*-values.

#### Correlations With Behavior

We were also interested in understanding if activation in any of our ROIs was related to literacy and/or age within the child groups. Only the child data were used for the correlation analysis, and we only performed correlations for ROIs for which the prior ANOVA analyses indicated were not yet adult-like: LmIPS, LvPG. Peak percent BOLD signal change and the literacy score were z-scored. We performed Pearson correlations analyses to assess the relationship between activation in each ROI and literacy as well as age. We note that the literacy composite score and the four independent assessments that comprise it were highly correlated with age: WJ-IV Letter-Word Identification (*r* = 0.793), WJ-IV Spelling (*r* = 0.816), WJ-IV Word Attack (*r* = 0.854), WJ-IV Spelling of Sounds (*r* = 0.870), literacy composite score (*r* = 0.857), all *p*s > 0.05. We, therefore, performed partial correlations analyses to assess the relationship between activation in each ROI and literacy, controlling for age. All *p*-values are reported as uncorrected *p*-values; however, all correlation analyses survived Bonferonni correction, i.e., for the 2 ROI-literacy composite score correlations at a threshold of *p_bonferroni_* = 0.05/2 = 0.0025 and for the 4 ROI-assessment score correlations at a threshold of *p_bonferroni_* = 0.05/4 = 0.0125.

All statistical analyses were performed using IBM SPSS Statistics for Mac OSX, version 26.

## Results

### ROI Analyses

#### Ventral Temporal Cortex

##### Left Anterior Fusiform Gyrus

**FIGURE 3:**
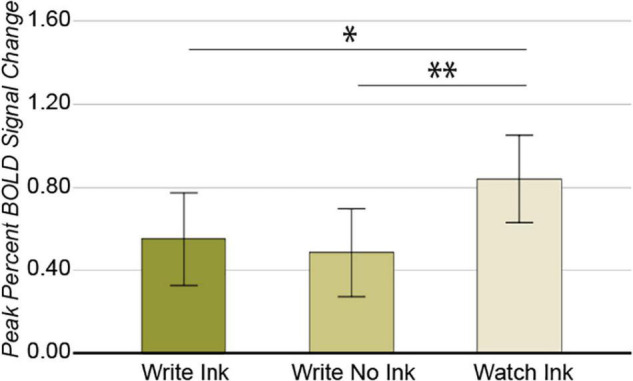
Left anterior fusiform gyrus. Main effect of CONDITION. Error bars represent 95% confidence intervals. ^∗∗^*p* < 0.01; ^∗^*p* < 0.05.

The 3 × 3 ANOVA in this region revealed a main effect of condition [*F*(2,76) = 4.194, *p* = 0.019, *post hoc* power 0.721; Figure 3]. The LaFuG response was greater during the Watch Ink (M = 0.834, SD = 0.713) condition than during the Write Ink (M = 0.553, SD = 0.705) and Write No Ink (M = 0.481, SD = 0.668) conditions [*t*(40) = 2.024, *p* = 0.050 and *t*(40) = 2.822, *p* = 0.007, respectively]. There was no difference between the Write Ink and Write No Ink conditions [*t*(40) = 0.534, *p* = 0.596]. The main effect of age group was not significant [*F*(2,38) = 1.713, *p* = 0.194, *post hoc* power 0.337], nor was the two-way interaction [*F*(4,76) = 1.698, *p* = 0.159, *post hoc* power 0.497].

##### Left Posterior Fusiform Gyrus

**FIGURE 4:**
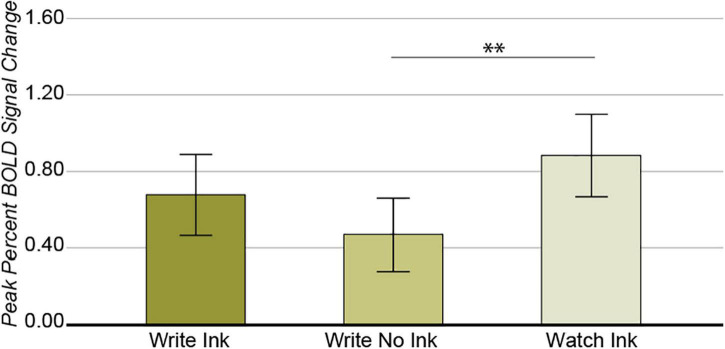
Left posterior fusiform gyrus. Main effect of CONDITION. Error bars represent 95% confidence intervals. ^∗∗^*p* < 0.01.

Again, we observed a significant main effect of condition in this region [*F*(2,74) = 5.052, *p* = 0.009, *post hoc* power 0.803; Figure 4], with greater response during the Watch Ink (M = 0.876, SD = 0.700) condition than Write No Ink (M = 0.426, SD = 0.641) condition [*t*(40) = 3.246, *p* = 0.002]. There was no difference between the Watch Ink and Write Ink (M = 0.677, SD = 0.641) conditions [*t*(39) = 1.548, *p* = 0.130] or the Write Ink and Write No Ink conditions [*t*(39) = 0.1.671, *p* = 0.103]. The main effect of age group was not significant [*F*(2,37) = 0.715, *p* = 0.496, *post hoc* power 0.162], nor was the two-way interaction [*F*(4,74) = 2.050, *p* = 0.096, *post hoc* power 0.586].

#### Parietal Cortex

##### Left Anterior Intraparietal Sulcus

**FIGURE 5:**
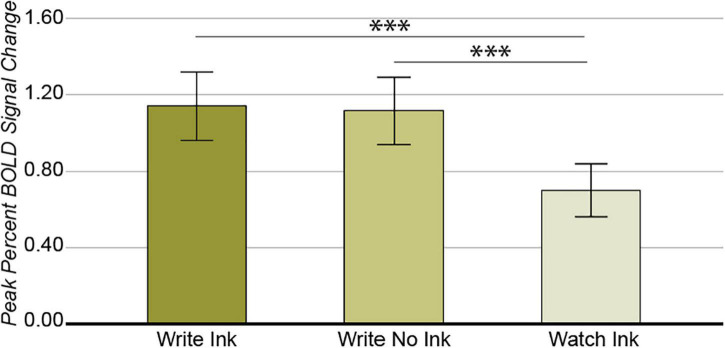
Left anterior intraparietal sulcus. Main effect of CONDITION. Error bars represent 95% confidence intervals. ^∗∗∗^*p* < 0.001.

As with the ventral temporal cortex, the main effect of condition was significant [*F*(2,74) = 11.851, *p* = 0.00003, *post hoc* power 0.993; Figure 5]. The LaIPS response was greater during the Write Ink (M = 1.14, SD = 0.556) and Write No Ink (M = 1.12, SD = 0.541) conditions than during the Watch Ink (M = 0.506, SD = 0.441) condition [*t*(40) = 4.622, *p* = 0.00004 and *t*(39) = 4.411, *p* = 0.00008, respectively]. There was no difference between the Write Ink and Write No Ink conditions [*t*(39) = 0.220, *p* = 0.827]. The main effect of age group was not significant [*F*(2,37) = 1.496, *p* = 0.237, *post hoc* power 0.298], nor was the two-way interaction [*F*(4,74) = 0.806, *p* = 0.526, *post hoc* power 0.246].

##### Left Middle Intraparietal Sulcus

**FIGURE 6:**
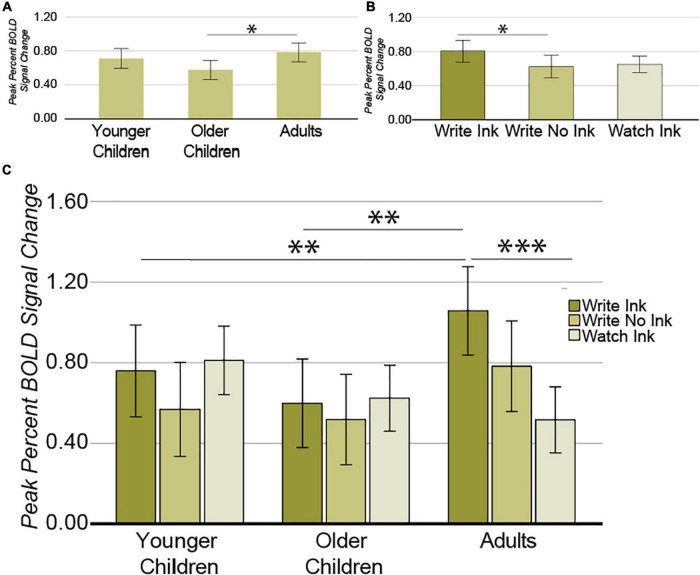
Left middle intraparietal sulcus. **(A)** Main effect of AGE GROUP. **(B)** Main effect of CONDITION. **(C)** Interaction between AGE GROUP and CONDITION. Error bars represent 95% confidence intervals. ^∗∗∗^*p* < 0.001; ^∗∗^*p* < 0.01; ^∗^*p* < 0.05.

A different pattern of results emerged from this region compared with our other ROIs. First, the main effect of age group was significant [*F*(2,38) = 3.543, *p* = 0.039, *post hoc* power 0.624] (Figure 6A). A *post hoc* independent samples *t*-tests revealed that the difference between adults (M = 0.785, SD = 0.220) and older children (M = 0.580, SD = 0.184) was significant [*t*(26) = 2.678, *p* = 0.013] but that the difference between older children and younger children was not [*t*(25) = 1.731, *p* = 0.096] (*p_bonferroni_* = 0.05/2 = 0.025). Second, the main effect of condition was marginally significant [*F*(2,76) = 2.632, *p* = 0.079, *post hoc* power 0.509] (Figure 6B). The LmIPS response was greater during the Write Ink condition (M = 0.806, SD = 0.441) than during the Write No Ink condition (M = 0.624, SD = 0.421) [*t*(40) = 2.033, *p* = 0.049]. The difference between the Write Ink condition and the Watch Ink condition (M = 0.647, SD = 0.319) was marginally significant [*t*(40) = 1.786, *p* = 0.082]. The difference between Write No Ink and Watch Ink was not significant [*t*(40) = 0.256, *p* = 0.800].

Most importantly, however, the ANOVA revealed a significant two-way interaction between age group and condition [*F*(2,76) = 2.926, *p* = 0.026, *post hoc* power 0.762; Figure 6C]. The LmIPS response differed as a function of condition in the adults [*F*(2,26) = 7.719, *p* = 0.002], but not in the younger children [*F*(2,24) = 1.071, *p* = 0.359], or in the older children [*F*(2,26) = 0.358, *p* = 0.703]. In adults, the LmIPS response decreased linearly from Write Ink (M = 1.057, SD = 0.333) to Write No Ink (M = 0.783, SD = 0.471) to Watch Ink (M = 0.517, SD = 0.278) [*F*(1,13) = 48.359, *p* = 0.00001]. The LmIPS response during Write Ink was greater than during Watch Ink in adults [*t*(13) = 6.954, *p* = 0.00001]. The LmIPS response during Write Ink was greater in adults than in the older children (M = 0.598, SD = 0.437) and greater than in the younger children as well (M = 0.759, SD = 0.439) [*t*(26) = 3.122, *p* = 0.004 and *t*(25) = 1.994, *p* = 0.057, respectively].

##### Left Posterior Intraparietal Sulcus

The ANOVA from this region demonstrated no significant main effects [condition: *F*(2,74) = 2.122, *p* = 0.127, *post hoc* power 0.422; age group: *F*(2,37) = 0.032, *p* = 0.968, *post hoc* power 0.054] and no significant interaction between the factors [*F*(4,74) = 1.382, *p* = 0.248, *post hoc* power 0.410].

#### Frontal Motor Cortex

##### Left Dorsal precentral Gyrus

**FIGURE 7:**
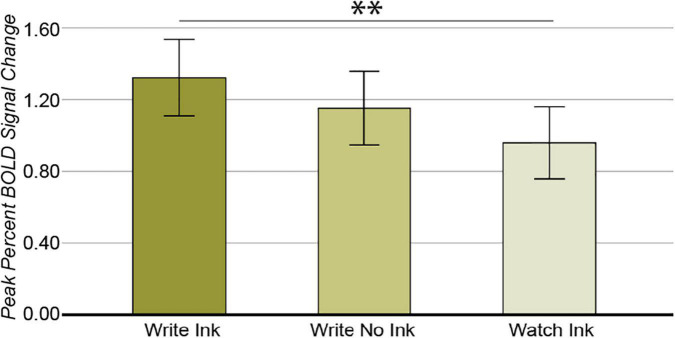
Left dorsal precentral gyrus. Main effect of CONDITION. Error bars represent 95% confidence intervals. ^∗∗^*p* < 0.01.

The ANOVA from this region revealed a main effect of condition [*F*(2,74) = 4.324, *p* = 0.017, *post hoc* power 0.735; Figure 7]. The LdPG response was greater during the Write Ink (M = 1.327, SD = 0.665) condition than during the Watch Ink (M = 0.960, SD = 0.665) condition [*t*(39) = 2.973, *p* = 0.005]. There was no difference between the Write Ink and Write No Ink (M = 1.152, SD = 0.626) conditions [*t*(39) = 1.264, *p* = 0.214]. There was no difference between the Write No Ink and Watch Ink conditions [*t*(39) = 1.667, *p* = 0.104]. The main effect of age group was not significant [*F*(2,37) = 1.035, *p* = 0.365, *post hoc* power 0.217], nor was the interaction [*F*(4,74) = 1.842, *p* = 0.130, *post hoc* power 0.534].

##### Left Ventral Precentral Gyrus

**FIGURE 8:**
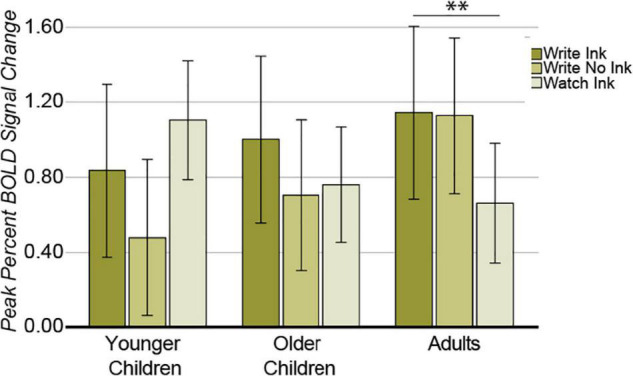
Left ventral precentral gyrus. Interaction between AGE GROUP and CONDITION. Error bars represent 95% confidence intervals. ^∗∗^*p* < 0.01.

In this region, the two main effects were not significant [condition: *F*(2,74) = 1.325, *p* = 0.272, *post hoc* power 0.278; age group: *F*(2,37) = 0.468, *p* = 0.630, *post hoc* power 0.121]. The two-way interaction was significant [*F*(4,74) = 2.638, *p* = 0.041, *post hoc* power 0.711; Figure 8]. The LvPG response differed among conditions in the adults [*F*(2,24) = 11.998, *p* = 0.0002], but not in the younger children, [*F*(2,24) = 1.880, *p* = 0.174] or in the older children [*F*(2,26) = 0.845, *p* = 0.441]. In the adults, the LvPG response was greater during the Write Ink condition (M = 1.145, SD = 0.541) than during the Watch Ink condition (M = 0.643, SD = 0.499) [*t*(12) = 4.633, *p* = 0.001]. There was no difference between the Write Ink and Write No Ink (M = 1.128, SD = 0.524) conditions [*t*(12) = 0.138, *p* = 0.892]. There was no difference between the Write No Ink and Watch Ink conditions [*t*(13) = 1.527, *p* = 0.151].

### Correlations With Behavior

#### Age

We evaluated the Pearson correlation between age and activation during the Write Ink condition using only the child data. We performed this correlation in only the LmIPS and LvPG given that these were the only ROIs that demonstrated an interaction between age and condition. We combined the two child age groups, i.e., younger and older, into one group because we did not observe any differences between these two age groups in the prior analyses. All correlations between activation and age were not significant, all *p*s > 0.05.

#### Literacy

We evaluated the partial correlation between a literacy composite score and activation during the Write Ink condition, controlling for age (see section “Materials and Methods: Statistical Analyses: Correlations with Behavior”). We used only the child data because we were concerned with the relationship between the neural system supporting handwriting and literacy during early reading development. We performed this analysis in the Write Ink condition only because we were concerned with the relationship between neural response during handwriting and literacy, while we had no specific hypotheses concerning relationships with the neural response in our control conditions.

**FIGURE 9:**
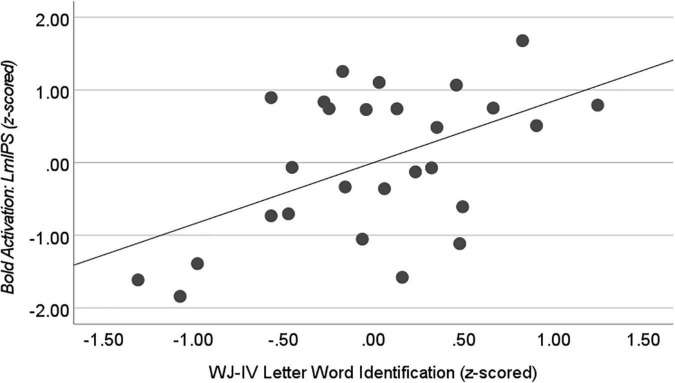
Correlation with WJ-IV Letter Word Identification during Write Ink. Activation in the left middle intraparietal sulcus (LmIPS) correlated with performance on the WJ-IV Letter Word Identification in the child participants after controlling for age (*r* = 0.515, *n* = 27, *p* = 0.007).

We found a positive correlation between Literacy and LmIPS activation during Write Ink (*r* = 0.447, *n* = 27, *p* = 0.022). As literacy increased, activation in the LmIPS increased (Figure 9). The correlation between Literacy and LvPG activation during Write Ink was not significant (*r* = 0.237, *n* = 27, *p* = 0.244).

The literacy composite score is an average of four independent assessments related to early reading development. We performed an additional exploratory analysis in the ROIs where activation during Write Ink correlated with the composite literacy score. We correlated each individual assessment score with activation to determine if there were certain aspects of literacy development that were related to activation in LmIPS in our child sample. We found a significant correlation between WJ-IV Achievement: Letter-Word Identification and LmIPS activation (*r* = 0.515, *n* = 27, *p* = 0.007). No other individual assessment correlated with LmIPS activation during Write Ink, all *p*s > 0.05.

## Discussion

Our goal with the present work was to provide information concerning the development of the neural system supporting handwriting in young children in the early stages of learning to write. We compared functional activation in 5–8-year-old children during fMRI scanning to activation in adults during handwriting and two sensorimotor control tasks. The sensorimotor control tasks allowed us to assess to what degree cortical regions associated with handwriting were related to the sensory (in this case visual) and/or motor components of handwriting. We focused our analyses on regions that have been shown to be related to the sensorimotor element of handwriting and tested whether the involvement of these regions in the sensorimotor element differed among young children and adults. Our results demonstrated that frontal motor and anterior parietal regions responded preferentially for the motor component while ventral regions responded preferentially for the visual component in both children and adults. We found a significant difference between children and adults in activation during handwriting in the left middle intraparietal sulcus (LmIPS) and left ventral precentral gyrus (LvPG), suggesting that the dorsal neural system that supports handwriting is still developing in children ages 5–8 years of age. Furthermore, we found that parietal and frontal activation correlated with a literacy composite score in our child sample, suggesting that the individual differences in parietal and frontal responses during handwriting were related to individual differences in emerging literacy skills. Our results are consistent with literature suggesting a prolonged developmental trajectory for parietal function relative to ventral-temporal function and suggest that the neural system that supports handwriting is related to early reading development.

### Adult Activation During Handwriting: Consistency With Prior Work

Activation during handwriting in the adult group was consistent with prior literature that has demonstrated a gradient of functional selectivity where dorsal/anterior brain regions are related to motor processing while ventral/posterior brain regions are related to visual processing (James and Gauthier, 2006; Planton et al., 2013, 2017; Vinci-Booher et al., 2019). In the current study, frontal and anterior parietal ROI responses were greatest during motor actions (Write Ink and Write No Ink) while ventral-temporal ROI responses were greatest in the visual-only condition (Watch Ink) in both child and adult participants.

The frontal motor ROI responses were greatest in the motor conditions relative to the visual-only condition. For both the dorsal and ventral precentral gyrus ROIs (i.e., LdPG and LvPG), activation was greater during Write Ink compared to Watch Ink and was not different during Write Ink than during Write No Ink. In the LdPG, activation was greater during Write Ink than during Watch Ink with no significant difference between Write Ink and Write No Ink. The same result was observed in the LvPG in the adult group. These results suggest that activation in the frontal motor cortex, specifically in the precentral gyrus, during adult-like handwriting is related to the execution of the motor action, consistent with prior works on handwriting (Planton et al., 2013; Kadmon Harpaz et al., 2014; Longcamp et al., 2014; Yuan and Brown, 2014; Vinci-Booher et al., 2019) and the literature on frontal motor cortex more broadly (Schieber, 2001; Graziano, 2006; Meier et al., 2008).

The parietal ROI responses demonstrated an anterior-posterior gradient along the IPS with anterior regions being related to motor processing, similar to prior works (Purcell et al., 2011; Thaler and Goodale, 2011; Planton et al., 2013; Kadmon Harpaz et al., 2014; Longcamp et al., 2014; Yuan and Brown, 2014; Haar et al., 2015; Vinci-Booher et al., 2019; Vinci-Booher and James, 2020b). The anterior IPS response was greater during Write Ink and Write No Ink compared to Watch Ink and the middle IPS activation decreased linearly from Write Ink to Write No Ink to Watch Ink in the adults. The posterior IPS response, however, was not significantly different across conditions, suggesting that posterior IPS does not necessarily prefer a motor condition to a visual-only condition. This suggests that the more anterior ROI was more strongly driven by the motor component relative to the posterior ROI, similar to prior work in adults using the MRItab (Vinci-Booher and James, 2020b).

The ventral-temporal ROI responses demonstrated a preference for the visual-only condition relative to the motor conditions; their responses were greater during Watch Ink than during Write Ink and Write No Ink. This result is consistent with literature demonstrating that the ventral-temporal cortex is largely involved in perceptual processing of sensory information, particularly visual information (Mishkin et al., 1983; Goodale and Milner, 1992). However, this result is also inconsistent with this literature because we found no significant difference in ventral-temporal response between the Write Ink and Write No Ink conditions. A difference between the Write Ink and Write No Ink conditions in ventral-temporal response would be expected because the letter produced during handwriting in the Write Ink condition is visually available to the participant while it is not available in the Write No Ink condition. Indeed, one of our prior studies demonstrated that the ventral-temporal cortex response was sensitive to visual images of letters that occur during handwriting, reporting a greater ventral-temporal response during Write Ink than during Write No Ink (Vinci-Booher et al., 2019). This apparent inconsistency can be rectified by noting that the experimental set-up in prior works does not allow participants to see their hands during handwriting, rendering the Write No Ink condition void of any letter-related visual input (e.g., Tam et al., 2011). The experimental set-up in the current study, however, allowed participants to see their hands during handwriting; in other words, participants watched their hand make the motions necessary to produce a written letter during the Write No Ink condition in the current study. Thus, our findings, in the context of the prior work discussed, suggest that activation in the ventral-temporal cortex during handwriting is sensitive to both visual input of the letter-form and also the visual input of one’s hand creating that form.

### Children Display Adult-Like Activation in Ventral-Temporal Cortex but Not Parietal or Frontal Cortex During Handwriting

Our results demonstrated that the magnitude of response in the ventral-temporal cortex was at an adult-like level by 4.5 years of age during handwriting; however, it is important to note that the visual processes being performed in the ventral-temporal cortex likely continue to change past 4.5 years of age. For example, prior work using this same child participant cohort demonstrated that the ventral-temporal cortex response was greater when children saw their own handwritten letters than when they saw typed letters and that the reverse was true by 6.5 years of age (Vinci-Booher and James, 2020a). Additionally, we note that neural processing for visual perception of common objects, such as faces and places, in the ventral-temporal cortex develops throughout childhood and adolescence (Golarai et al., 2007; Scherf et al., 2007) as does processing for written words (Centanni et al., 2017) but see Dehaene-Lambertz et al. (2018). In the context of prior works, our results suggest that ventral-temporal cortex may already be responding during handwriting, as it does in adults, but likely still undergoes changes in sensitivity to visual stimuli at later ages.

Our results clearly demonstrated that handwriting-related function in the parietal cortex was still developing in our child sample. We found a significant difference in activation magnitude among our age groups in the left middle intraparietal sulcus (LmIPS). Activation in the LmIPS was dependent on an interaction between age group and condition such that its response was greatest during Write Ink in the adult group. In adults, the LmIPS response was greater during Write Ink when compared to Write No Ink and Watch Ink while there were no significant differences among these conditions in either child group.

Prior work in adult participants has demonstrated that activation along the left intraparietal sulcus is more closely associated with handwriting than drawing, indicating that one of the crucial differences between the neural system that supports these activities is parietal function (Yuan and Brown, 2014, 2015; Ose Askvik et al., 2020). Perhaps the most notable differences between handwriting and drawing are, first, that handwriting is more strongly associated with language than drawing and, second, that handwriting becomes an over-practiced skill whereas drawing typically remains under-practiced. It is unlikely that the condition and age group interaction that we observed in LmIPS activation was related to the association of handwriting with language because our conditions specifically manipulated the sensorimotor aspects of handwriting, leaving the language association in each condition. Therefore, the condition differences observed in LmIPS activity in adults were not likely due to an association with language in the Write Ink condition that was not present in the Write No Ink and Watch Ink conditions. We interpret the greater activation in LmIPS during Write Ink in adults compared to children to be associated with the performance of an over-practiced task, a task that would not be over-practiced in 5–8-year-old children. Although children begin to learn the difference between handwriting and drawing as young as 3 years old based on behavioral measures (Treiman and Yin, 2011; Otake et al., 2017), their productions are far from being over-practiced and our results suggest that during handwriting children are likely relying on a neural system similar to the neural system used for drawing in adults.

The neural response during handwriting in the left frontal motor cortex also exhibited developmental differences, but only for the ventral precentral gyrus (LvPG) and not the dorsal precentral gyrus (LdPG) ROI. In the LvPG, activation in adults was greater during Write Ink than during Watch Ink, but there was no difference between these two conditions in either child group. The response in adults suggests that the LvPG is associated with the execution of the motor action, similar to the LdPG. However, unlike the LdPG, the LvPG is not yet adult-like in our child sample, suggesting that ventral portions of the precentral gyrus undergo a more prolonged developmental trajectory than dorsal portions of the precentral gyrus.

### Activation in LpIPS and LdPG Correlates With Literacy in Children

Our results demonstrated a significant correlation between a literacy composite score and activation in the left posterior intraparietal sulcus (LpIPS) and between the same literacy composite score and activation in the left dorsal precentral gyrus (LdPG). The composite literacy score was created by averaging across several literacy-related subtests of the WJ-IV, including subtests that assessed reading real and non-real words as well as spelling real and non-real words. When we tested for correlations between the subtests that were used in the composite score, we found that the LpIPS and LdPG correlations were driven by the children’s scores on the Letter-Word Identification subtask. The Letter-Word Identification was the subtest that assessed reading real words and, for younger children, often includes only letter identification items. Our results, therefore, suggest that activation in the LpIPS and LdPG during handwriting is related to letter recognition and word reading ability.

A substantial line of research suggests that learning to read is accompanied by changes in ventral-temporal function during passive word reading tasks (Centanni et al., 2018; Chyl et al., 2018; Dehaene-Lambertz et al., 2018; Lerma-Usabiaga et al., 2018; Nordt et al., 2018; Kubota et al., 2019; Brem et al., 2020; Liebig et al., 2021). We, therefore, had expected to find a correlation between literacy and ventral-temporal activation during handwriting; however, we instead found a correlation between literacy and dorsal motor activation during handwriting. This suggests that activation in ventral-temporal and dorsal motor cortex is different during passive reading tasks than it is during active production tasks, even when both tasks contain letters and words.

Studies that have investigated dorsal motor activity during handwriting suggest that activation in the dorsal motor cortex during handwriting may be related to letter recognition, similar to our results. One set of studies investigated activation in dorsal motor regions, specifically the LdPG and LaIPS, in adults as they wrote individual letters of the alphabet (i.e., a, s) in different letter-forms (i.e., a A) (Rapp and Dufor, 2011; Dufor and Rapp, 2013). Results demonstrated that the response in these dorsal motor regions during handwriting was related to the identity of the letter (i.e., a vs. s) and not to the different letter-forms (i.e., a vs. A). This result suggests that activation in the dorsal motor cortex during handwriting may be related to letter recognition and, remarkably, may not be related to the specification of the hand movements required to produce a letter-form. Such a result fits nicely with a large body of work that has interpreted activation in dorsal motor regions during passive letter perception as signifying the involvement of the motor system in letter recognition (Longcamp et al., 2003, 2005, 2006, 2008; James and Gauthier, 2006; James and Atwood, 2009; James, 2010, 2017; James and Engelhardt, 2012; Vinci-Booher and James, 2020a).

## Conclusion

The current study is the first study to investigate the neural correlates of handwriting in typically developing children under the age of 8 years old. Prior work has suggested that the adult neural system supporting handwriting is already in place by 8 years of age. Here, we demonstrated that parietal and frontal motor regions are not yet adult-like by 5 years of age, suggesting that the neural system supporting handwriting changes during the early elementary school years. Further, we found a positive correlation between dorsal neural activity and early literacy skills. Our results are consistent with the broad developmental trend whereby function in ventral-temporal cortex resembles adult function earlier than function in the parietal cortex when examined past the age of 2 years old.

## Data Availability Statement

The original contributions presented in the study are included in the article/supplementary material, further inquiries can be directed to the corresponding author/s.

## Ethics Statement

The studies involving human participants were reviewed and approved by the Indiana University Institutional Review Board. Written informed consent to participate in this study was provided by the participants’ legal guardian/next of kin. Children 7 years and older provided written assent.

## Author Contributions

SV-B contributed to all aspects of the manuscript, including the original conception of the study, the design, data collection, analyses, writing the original draft of the manuscript, and revisions. KJ contributed to conceptual development of the work, the design, data collection, and the writing of the manuscript.

## Conflict of Interest

This report makes use of a patented MRI-compatible device (US Patent No. 62/370, 372). The patent is owned by the Trustees of Indiana University (Inventors: Sturgeon, Shroyer, SV-B, and KJ).

## Publisher’s Note

All claims expressed in this article are solely those of the authors and do not necessarily represent those of their affiliated organizations, or those of the publisher, the editors and the reviewers. Any product that may be evaluated in this article, or claim that may be made by its manufacturer, is not guaranteed or endorsed by the publisher.
